# Mode of delivery and other risk factors for *Escherichia coli* infections in very low birth weight infants

**DOI:** 10.1186/1471-2431-14-274

**Published:** 2014-10-18

**Authors:** Agnieszka Chmielarczyk, Jadwiga Wójkowska-Mach, Dorota Romaniszyn, Paweł Adamski, Ewa Helwich, Ryszard Lauterbach, Monika Pobiega, Maria Borszewska-Kornacka, Ewa Gulczyńska, Agnieszka Kordek, Piotr B Heczko

**Affiliations:** Department of Microbiology, Jagiellonian University Medical College, 18 Czysta Street, 31-121 Krakow, Poland; Institute of Nature Conservation, Polish Academy of Sciences, Krakow, Poland; Clinic of Neonatology and Intensive Neonatal Care, Institute of Mother and Child, Warsaw, Poland; Clinic of Neonatology, Jagiellonian University Medical College, Krakow, Poland; Clinic of Neonatology and Intensive Neonatal Care, Warsaw Medical University, Warsaw, Poland; Clinic of Neonatology, Polish Mothers’ Memorial Hospital-Research Institute, Lodz, Poland; Department of Neonatal Diseases, Pomeranian Medical University, Szczecin, Poland

**Keywords:** Very low birth weight, Infections, *Escherichia coli*, Surveillance

## Abstract

**Background:**

Infections in newborns remain one of the most significant problems in modern medicine. *Escherichia coli* is an important cause of neonatal bloodstream and respiratory tract infections and is associated with high mortality. The aim of our study was to investigate the epidemiology of *E. coli* infection in Polish neonatal intensive care units (NICUs) and resistance to antibiotics, with particular reference to the safety of very low birth weight infants.

**Methods:**

Continuous prospective infection surveillance was conducted in 2009–2012 in five NICUs, including 1,768 newborns whose birth weight was <1.5 kg. *Escherichia coli* isolates from different diagnostic specimens including blood, tracheal/bronchial secretions and others were collected. All isolates were tested using disk diffusion antimicrobial susceptibility methods. Pulsed-field gel electrophoresis was used to determine the possible horizontal transfer of *E. coli* among patients.

**Results:**

The incidence of *E. coli* infections was 5.4% and 2.0/1,000 patient-days. The occurrence of *E. coli* infections depended significantly on the NICU and varied between 3.9% and 17.9%. Multivariate analysis that took into account the combined effect of demographic data (gender, gestational age and birth weight) and place of birth showed that only the place of hospitalisation had a significant effect on the *E. coli* infection risk. The highest levels of resistance among all *E. coli* isolates were observed against ampicillin (88.8%) and amoxicillin/clavulanic acid (62.2%). Among *E. coli* isolates, 17.7% were classified as multidrug resistant. *Escherichia coli* isolates showed different pulsotypes and dominant epidemic clones were not detected.

**Conclusions:**

Our data indicate that antibiotic prophylaxis in the presence of symptoms such as chorioamnionitis and premature rupture of membranes did not help reduce the risk of *E. coli* infection. Multivariate analysis demonstrated only one significant risk factor for *E. coli* infection among infants with a birth weight <1.5 kg, that is, the impact of the NICU, it means that both neonatal care and care during pregnancy and labour were found to be significant.

## Background

Infections in newborns, particularly those with very low birth weight of <1.5 kg, remain one of the most significant problems in modern medicine. *Escherichia coli* is an important cause of neonatal bloodstream and respiratory tract infections and is associated with high mortality [1, 2].

In late-onset infection, *E. coli* may be acquired from the mother or the environment and may enter the bloodstream from the urinary or gastrointestinal tract [3]. Several studies have demonstrated a high rate of transmission of *E. coli* within families and households [4, 5]. *Escherichia coli* species comprise commensal strains, intestinal pathogenic strains and others that cause infections outside the gastrointestinal system, such as extra-intestinal pathogenic *E. coli*. Extra-intestinal infections due to *E. coli* are common in all age groups and include bacteremia, urinary tract infection, meningitis (mostly in neonates), nosocomial pneumonia and others [6].

Cephalosporin, fluoroquinolone and trimethoprim-sulfamethoxazole are widely used to treat infections caused by *E. coli* and resistance to these agents is responsible for treatment failure [7]. The development of multidrug resistance (MDR) among *E. coli* isolates is also of concern [8].

Enterobacteriaceae-producing extended-spectrum β-lactamases (ESBLs) have emerged as serious pathogens in hospitals and have been increasingly implicated in nosocomial outbreaks in neonatal intensive care units (NICUs). They have important therapeutic implications as they exhibit resistance to several antimicrobial agents, including third-generation cephalosporins, extended-spectrum penicillin and monobactam. Carbapenems and cephamycins represent the only classes of antibiotics active against ESBLs [9].

*Escherichia coli* has been reported as one of the major causes of neonatal infections that may cause high morbidity and mortality [10]. Potential risk factors of *E. coli* neonatal infections have also been analysed.

Considering the increasing antimicrobial resistance and mortality in NICUs, there is an urgent need to understand the epidemiology and risk factors of *E. coli* infections. Therefore, the aim of our study was to investigate the epidemiology of infections caused by *E. coli* in five Polish NICUs, resistance to antibiotics and potential risk factors that may contribute to such infections (*E. coli* infections were compared with infections caused by other bacteria). We also considered the possibility of using data on *E. coli* infection in assessing the quality of perinatal care. All these data may contribute to infection prevention and better control, and the routes of transmission of *E. coli* were also evaluated.

## Methods

### Ethics

The Polish Neonatal Surveillance Network (PNSN), whose director is Prof. Ewa Helwich from the Institute of Mother and Child in Warsaw, covered six Neonatal Clinics within the territory of Poland: Clinic of Neonatology and Intensive Neonatal Care, Institute of Mother and Child, Warsaw, Clinic of Neonatology, Jagiellonian University Medical College, Krakow, Clinic of Neonatology and Intensive Neonatal Care, Warsaw Medical University, Warsaw, Clinic of Neonatology, Polish Mothers’ Memorial Hospital-Research Institute, Lodz, Department of Neonatal Diseases, Pomeranian Medical University, Szczecin and Gynaecology and Obstetrics Hospital Medical University Poznan. The Polish Neonatal Surveillance Network was established on 6th of September 2007 (Agreement; see attachment). The Chair of Microbiology cooperated within the program of the PNSN. Utilization of data collected in the PNSN for scientific purposes was approved by the Bioethics Committee of Jagiellonian University Medical College (Chairperson Prof. Piotr Thor) – no. KBET/221/B/2011 ) on 27 October 2011 (Appropoval; see attachement). All data entered into the electronic database and analysed during this study were previously anonymised and de-identified.

### Study population

A prospective surveillance of infections was conducted between 1 January 2009 and 31 December 2012 in five from six NICUs that participated in the activities of the PNSN.

The study covered 1,768 newborns. All episodes of infection were subjected to registration, regardless of the time of occurrence of the first symptoms. Case patients were defined according to Gastmeier et al. [11], with modifications for neonates with birth weight <1.5 kg. Early-onset infection (EOI) was defined as infection diagnosed within 3 days of delivery. The occurrence of chorioamnionitis was based on clinical data (all cases) and histopathology examination of the placenta (~65% of cases).

### Bacterial isolates

Various diagnostic specimens including blood, and tracheal/bronchial secretions were collected for culture and assessment of the microbial aetiology of infections. Altogether, 96 *E. coli* strains were isolated, and the present study covered 90 isolates (six were not stored). Isolates were identified by the automated identification system (VITEK 2; bioMérieux, Warsaw, Poland). Isolates from other infections were also analysed. Aetiological factors of other infections were: other Gram-negative bacteria, Gram-positive bacteria, atypical bacteria and yeast.

### Antimicrobial susceptibility

All isolates were tested using disk diffusion antimicrobial susceptibility methods on Mueller–Hinton agar plates according to the current EUCAST guidelines (European Committee on Antimicrobial Susceptibility Testing. Clinical breakpoint tables v. 3.1; http://www.eucast.org v.3.1, accessed: 11.02.2013). ESBL activity was detected with a modified double disk synergy test using a combination of cefotaxime, ceftazidime, cefepime and aztreonam discs, placed 20 mm apart around the disc containing amoxicillin/clavulanic acid [12].

### Pulsed-field gel electrophoresis (PFGE)

PFGE was used to determine the possible horizontal transfer of *E. coli* strains among patients. All isolates were analysed using the standardized PFGE protocol developed at the Centers for Disease Control and Prevention by the PulseNet program http://ttp://www.cdc.gov/pulsenet/pathogens/ecoli.html (accessed: 11.02.2013). Genomic DNA was digested with 10 U *Xba*I (ThermoScientific, ABO, Gdansk, Poland). The resulting DNA fingerprinting was analysed using the CHEF III PFGE system (BioRad, Warsaw, Poland) in 0.5 Tris–borate–EDTA buffer at 14°C at 6 V for 20 h with a ramped pulse time of 2.2–54.2 s. The GelCompar (Applied Maths) was used for cluster analysis using the Dice coefficient and the unweighted pair group method with arithmetic mean.

### Risk factors

Several potential risk factors were compared according to infections caused by *E. coli* and by other strains: birth weight, gestational age, CRIB, Apgar score, sex, type of birth, feeding patterns (breastfeeding, trophic feeding, or parenteral nutrition), perinatal antibiotic prophylaxis, prolonged premature rupture of membranes (PROM).

### Statistical analysis

The influence of type of care and sociodemographic characteristics on the epidemiology of *E. coli*/other infection was analysed with several statistical techniques, depending on type and distribution of analysed variables. The relation between probability of *E. coli* and continuous parameters (age, length of stay) was based on simple analysis of variance (ANOVA). If the distribution of continuous variables significantly differed from normality, the nonparametric alternative to ANOVA, the Kruskal–Wallis test, was used. For the contingency of nominal characters frequency test: Pearson’s chi-square (χ^2^) and likelihood ratio was used. The common influence of risk factors on *E. coli* identification was analysed with a generalised linear model. Because of the categorical character of the effect and combined — numerical as well as categorical — types of the predictors, the model was constructed for binominal distribution of dependent variables and logit-linked function. A p value <0.05 was considered significant. All analyses were performed using JMP version 9.3 (SAS Institute Inc., Cary, NC, USA).

## Results

### Non-*E. coli*infections

Neonates with infections of non-*E. coli* aetiology were characterised by better general condition at birth (Table 1) than neonates with *E. coli* infections. Significantly fewer infants had adverse perinatal outcomes, such as PROM or chorioamnionitis, while significantly more newborns were delivered by caesarean section. The fatality rate of newborns with non-*E. coli* infections was two times lower than in those with *E. coli* infections.

**Table 1:** **The characteristics of newborns with symptoms of infections and** ***E. coli*** **infections**

	Infants without infections [N = 890]		Infants with other infections [N = 782]		OR	95% CI	P*-value	Infants with ***E. coli***-infections [N = 96]		OR	95% CI	P**-value
Risk factors	Average	95% CI	Average	95% CI				Average	95% CI			
Birth weight	1091.4	[1072.4, 1110.4]	991.1	[971.9,1010.2]	NA		<0.0001	909.1	[858.6, 959.6]	NA		<0.0001
Gestational age	29.1	[28.8, 29.3]	28.0	[27.8, 28.2]	NA		<0.0001	27.0	[26.5, 27.6]	NA		<0.0001
CRIB	4.5	[4.1, 5.0]	4.4	[4.0, 4.8]	NA		-	5.5	[4.1, 6.8]	NA		-
Apgar (1 min.)	5.5	[5.3, 5.6]	5.3	[5.2, 5.4]	NA		<0.0001	4.8	[4.4, 5.2]	NA		<0.0001
Apgar (5 min.)	6.0	[5.9, 6.2]	6.0	[5.9, 6.2]	NA		-	5.7	[5.3, 6.1]	NA		<0.0001
Number and percentage of population												
Female gender	438	49.2	350	44.8	1.19	0.986-1.45	-	44	45.8	0.96	0.626-1.465	-
Caesarean section	731	82.1	624	79.8	1.03	0.803-1.326	0.0002	62	64.6	2.17	1.377-3.408	<0.0001
Breast feeding	64	7.2	44	5.6	1.29	0.874-.932	-	3	3.1	5482	0.563-6.071	-
Trophic feeding	295	33.1	310	39.6	0.75	0.618-0.922	0.008	42	43.8	0.84	0.550-1.295	<0.0001
Total parenteral nutrition	319	35.8	409	52.3	0.51	0.419-0.619	<0.0001	56	58.3	0.78	0.509-1.203	<0.0001
Perinatal antibiotic prophylaxis	335	37.6	344	44.0	0.77	0.632-0.935	0.009	46	47.9	0.85	0.558-1.305	<0.0001
Death of the patient	209	23.5	64	8.2	3.44	2.553-4.643	<0.0001	14	14.6	0.52	0.280-0.972	<0.0001
Infants born from deliveries where the following were diagnosed during the mother’s pregnancy:												
PROM	185	20.8	211	27.0	0.71	0.566-0.890	0.0008	33	34.4	0.71	0.449-1.106	<0.0001
Chorioamnionitis	62	7.0	60	7.7	0.90	0.623-1.303	-	13	13.5	0.53	0.279-1.000	<0.0001

P* value: analysis of infants without infection versus those with non-*E. coli* infections.

P** value: analysis of infants with non-*E. coli* infections versus infants with *E. coli* infections.

CRIB, clinical risk index for babies.

### *Escherichia coli*infections

The incidence of *E. coli* infections was 5.4% and 2.0/1,000 patient-days. EOIs accounted for 12.2% of all infections and late-onset infections (LOIs) accounted for 10.5%. The incidence of EOIs was 1.7% and LOIs was 3.7%. The occurrence of *E. coli* infections (both EOI and LOI) depended significantly on the NICU (χ^2^ = 73,836; p < 0.0001) and varied between 3.9% and 17.9%. The most common *E. coli* infection was pneumonia (53.1%) and bloodstream infection (40.6%). *Escherichia coli* infections were diagnosed at day 17 on average (median: 12 days).

The mean length of hospitalisation (from birth to discharge, or until a weight of 1.8 kg was reached) of newborns with non-*E. coli* infections was significantly shorter: 48 versus 55 days (infants with *E. coli* infection).

The gestational age and birth weight of newborns with *E. coli* infections were lower than in those without infection or with other infections (Table 1). The other risk factors of *E. coli* infections were: low Apgar 1 and Apgar 5 scores, total parenteral nutrition, chorioamnionitis, or PROM diagnosed during pregnancy or delivery (Table 1).

Caesarean section reduced the risk of *E. coli* infection, although perinatal antibiotic prophylaxis (PAP) (no standardised protocol) did not reduce the risk of infection; that is, despite the use of PAP, *E. coli* infections were observed significantly more frequently (Table 1).

### Multivariate analysis

Multivariate analysis that took into account the combined effect of intrapartum antibiotic prophylaxis (IAP), PROM, chorioamnionitis and delivery mode (caesarean section) showed no significant effect on the risk of *E. coli* infection for factors other than the mode of delivery. However, multivariate analysis that took into account the combined effect of demographic data (sex, gestational age and birth weight) and place of birth (NICU) showed that only the place of hospitalisation had a significant effect on *E. coli* infection risk.

Finally, a multivariate analysis of maternal influences on IAP, PROM, amniotic inflammation, type of labour, gestational age, NICU and the likelihood of specific *E.coli* infections *(vs.* other infections) showed a significant statistical relationship with the complete model.

### Escherichia coli resistance and similarity of strains

Analysis of the isolates showed that the highest levels of resistance among all *E. coli* isolates were observed against ampicillin (88.8%) and amoxicillin/clavulanic acid (62.2%), trimethoprim/sulfamethoxazole (34.4%) and aztreonam (33.3%). The ESBL phenotype was found among 25 isolates (27.7%). Sixteen of the ESBL-positive strains (17.7%) were also reported as resistant to at least two other groups of antibiotics (fluoroquinolones, aminoglycosides or trimethoprim–sulfamethoxazole), which allows us to consider these strains as MDR organisms (Table 2).

**Table 2:** **Characteristics of ESBL-positive strains**

Isolate	Site of isolation	Resistance profile	MDR organism (yes/no)
**114**	Respiratory tract	Amp, Amc, Fep, Cxm, Atm, Tob, Cn, Sxt, C	Yes
**120**	Respiratory tract	Amp, Amc, Fep, Caz, Ctx, Cxm, Atm, Sxt, Cip, C	Yes
**124**	Respiratory tract	Amp, Amc, Fep, Caz, Ctx, Cxm, Atm, Tob, Cn, Sxt, Cip, C	Yes
**4S**	Respiratory tract	Amp, Amc, Fep, Caz, Ctx, Cxm, Atm, Tob, Cn, Ak	No
**110S**	Rectum	Amp, Amc, Caz, Ctx, Cxm, Atm, Dor	No
**227**	Respiratory tract	Amp, Amc, Fep, Ctx, Cxm, Atm	No
**197**	Amniotic fluid	Amp, Amc, Fep, Ctx, Cxm, Atm	No
**226**	Blood	Amp, Amc, Fep, Caz, Ctx, Cxm, Atm, Tob, Sxt, Cip	Yes
**7**	Respiratory tract	Amp, Amc, Fep, Caz, Ctx, Cxm, Atm, Tob	No
**13**	Respiratory tract	Amp, Amc, Ctx, Cxm, Atm	No
**118**	Respiratory tract	Amp, Amc, Caz, Ctx, Cxm, Atm, Tob, Sxt, Cip	Yes
**159**	Respiratory tract	Amp, Amc, Caz, Ctx, Cxm, Atm, Tob, Cn, Sxt, Cip,Tzp	Yes
**160**	Respiratory tract	Amp, Amc, Caz, Ctx, Cxm, Atm, Tob, Cn, Sxt, Cip,Tzp	Yes
**105S**	Respiratory tract	Amp, Amc, Ctx, Cxm, Atm, Tob, Cn, Ak, Sxt	Yes
**24 W**	Rectum	Amp, Amc, Fep, Caz, Ctx, Cxm, Atm, Ak, Sxt, Cip	Yes
**32 W**	Rectum	Amp, Amc, Fep, Ctx, Cxm, Atm, Tob, Cn, Ak, Sxt	Yes
**14**	Blood	Amp, Amc, Fep, Caz, Ctx, Cxm, Atm, Tob, Cip	Yes
**62**	Blood	Amp, Amc, Fep, Caz, Ctx, Cxm, Atm, Tob, Ak, Sxt, Cip	Yes
**166**	Blood	Amp, Amc, Caz, Ctx, Cxm, Atm, Tob, Sxt, Cip	Yes
**205**	Blood	Amp, Amc, Fep, Caz, Ctx, Cxm, Atm, Tob, Sxt, Cip	Yes
**3 W**	Blood	Amp, Fep, Caz, Ctx, Cxm, Atm, Tob, Cn, Ak	No
**9**	Urine	Amp, Amc, Fep, Ctx, Cxm, Atm	No
**15**	Urine	Amp, Amc, Fep, Ctx, Cxm, Atm	No
**42**	Respiratory tract	Amp, Amc, Fep, Caz, Ctx, Cxm, Atm, Tob, Ak, Sxt, Cip	Yes
**112**	Respiratory tract	Amp, Amc, Fep, Ctx, Cxm, Dor, Atm, Sxt, C	Yes

Amp, ampicillin; Amc, amoxicillin/clavulanic acid; Fep, cefepime; Ctx, cefotaxime; Caz, ceftazidime; Cxm, cefuroxime; Dor, doripenem; Atm, aztreonam; Tob, tobramycin; Ak, amikacin; Cn, gentamicin; Sxt, trimethoprim/sulfamethoxazole; C, chloramphenicol; Cip, ciprofloxacin; Tzp, piperacillin/tazobactam.

Resistance to ampicillin (p = 0.0375), amoxicillin/clavulanic acid, ceftazidime, cefotaxime, cefuroxime, cefepime, aztreonam (all p-values <0.0001), amikacin (p = 0.0012), gentamicin (p = 0.0016), tobramycin (p < 0.0001), trimethoprim/sulfamethoxazole (p = 0.0016) and ciprofloxacin (p < 0.0001) in a group of ESBL-positive strains was significantly higher than in ESBL-negative strains. Only five of the studied isolates studies were resistant to one of four tested carbapenems; three of these isolates belong to non-ESBL strains. Almost all *E. coli* strains (97.8%) were susceptible to tigecycline and piperacillin/tazobactam.

**Figure 1:**
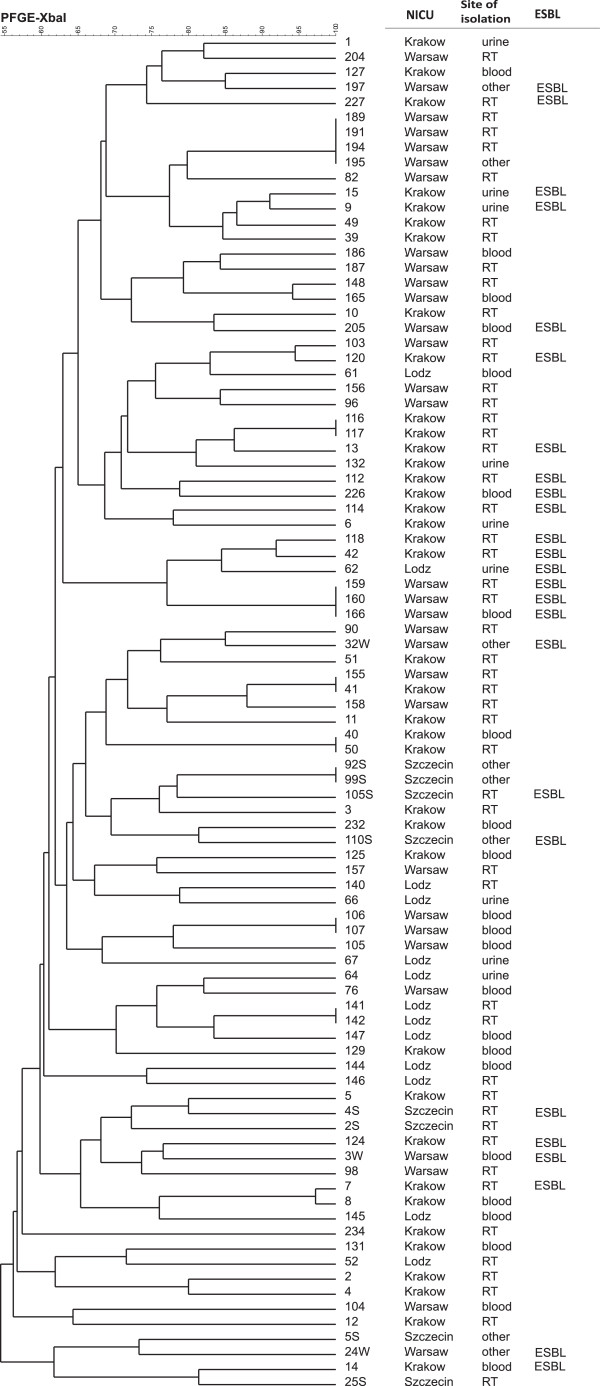
**Results of PFGE typing of** ***E. coli*** **isolates.**

*Escherichia coli* isolates showed that different pulsotypes and dominant epidemic clones were not detected. Cluster analysis based on PFGE of the 90 isolates showed 71 unique types, some of which were <70% similar, suggesting a genotypically variable population (Figure 1). Isolates that have identical pulsotypes usually were derived from the same patient (as in the case of 11 isolates) or were isolated from different patients of the same NICU in the same period of time (in the case of seven isolates). The location of the NICU and the site of the isolation did not appear to have a correlation in the cluster analysis.

## Discussion

Infections in newborns, particularly those with very low and extremely low birth weights of <1.5 kg, are a significant problem. *Escherichia coli* is responsible for a smaller fraction of neonatal infections than other microorganisms, but it is associated with the highest mortality [13].

Data collected by the European Antimicrobial Resistance Surveillance Network confirm that in Europe from 2002 to 2009 the occurrence of *E. coli* in bloodstream infections increased by more (71%) than *Staphylococcus aureus* (34%), which indicates the growing importance of *E. coli* in the epidemiology of infections [14] and the need for detailed molecular studies of these strains. Our study was performed to characterise the population of *E. coli* isolated from neonatal infections; mainly from pneumonia and bloodstream infections from Polish NICUs.

According to other data, *E. coli* causes about 9% of infections [15] or 5–13% of LOI in NICUs [15–17]. Additionally, in Polish NICUs, *E. coli* was the most frequently isolated pathogen in early-onset PNSN infections [18]. In recent years, *E. coli* infections in NICUs have become more prevalent, and it is believed that this is mainly owing to the implementation of PAP [19–21].

Other studies on *E. coli* have identified that low gestational age [22], duration of hospital stay [23] and use of antibiotics [24–26] are the risk factors of colonisation and infection among newborns. Our results support these data, and we hypothesise that another major risk factor may be maternal colonisation. According to Denkel et al., colonised mothers may be an important reservoir of *E. coli* and significant risk factors for *E. coli* colonisation, and consequently, *E. coli* infections of very low birth weight infants [27].

Unfortunately, screening of mothers was not performed in NICUs of PNSN, but our PFGE results support our hypothesis: the population of *E. coli* was divergent and no epidemic clones were identified. That is totally different than for other species such as methicillin-resistant *S. aureus* or *Klebsiella pneumoniae* [28].

The mode of delivery seems to be a protective factor against *E. coli* infections and also supports the hypothesis on maternal–neonatal transmission. About 17.9% of neonates without infections were born vaginally, compared with 35.4% of those with *E. coli* infection. The vagina is a significant reservoir of *E. coli*, which can be critical in vertical transmission of *E. coli* infection. Our results indicate that perinatal factors and vaginal transmission may also significantly affect the epidemiology of *E. coli* infection, including LOI, which could be important for control of hospital-acquired infections.

In our study, the fatality rate of *E. coli* infections was almost twice as high as that of infections of other aetiology. The reason for this was mainly the high pathogenicity of *E. coli*: the majority of tested strains belonged to one of two virulence groups (B2, 68.9%, or D, 17.8%) [6, 29]. Isolates from the B2 group had significantly more virulence genes than isolates from other groups; in particular, *fim*H, *sfa*, *ire*A, *fhu*A, *fyu*A and *fep*A genes were more likely to occur [29]. Similarly, 36.6% of strains belonged to the ST131 clone and showed a high level of virulence and resistance, which could play an important role in the epidemiological success of this sequence type. Clonal group ST131 contained slightly more than one-third of the studied isolates and had a link to ESBL production [29].

Among ESBL-positive isolates, which accounted for 27.7% of all isolates, higher levels of resistance to aminoglycosides and also ciprofloxacin and trimethoprim-sulfamethoxazole were observed compared with non-ESBL isolates. The higher level of resistance may contribute to the fact that such resistance is localised mainly on plasmids and may be easily transferred not only among *E. coli* isolates, but also among isolates belonging to other species. Using of one of the antibiotics (β-lactam, aminoglycoside, or fluoroquinolones) may lead to the co-selection of fluoroquinolone resistance by β-lactams or aminoglycosides, and *vice versa* β-lactams or aminoglycoside resistance by fluoroquinolones [30].

## Conclusions

Our study showed that there are some risk factors that should be considered during perinatal care. Routine screening of mothers and newborns for *E. coli* should be implemented in maternity care to decrease morbidity and mortality. Pre-partum hospitalisation for premature labour should be an indication for screening by rectal swabs, prenatally and on the day of birth. Unfortunately, the presented data indicate that PAP in the presence of symptoms such as chorioamnionitis and PROM did not help to reduce the risk of *E. coli* infections. Multivariate analysis demonstrated only one significant risk factor for *E. coli* infection among infants with a birth weight <1.5 kg, namely, the impact of the NICU.

### Consent

Electronic database created as the result of continuous prospective targeted surveillance of infections was used in the study.

Participation of hospitals in PNSN was voluntary and confidential. Utilization of data collected in PNSN for the scientific purpose was approved by the Bioethics Committee of Jagiellonian University Medical College – no. KBET/221/B/2011. All data entered into the electronic database and analyzed during preparing this article were previously anonymized and de-identified. Those data were obtained under routine treatment and diagnostic procedures performed during patients’ hospitalization. No additional samples were collected for testing. According to Polish law, utilization this kind of data for scientific purpose does not demand patients’ agreement or even information that data are collected in the database.

## Abbreviations

BSI: Bloodstream infection
EOI: Early-onset infection
ESBL: Extended-spectrum β-lactamase
IAP: Intrapartum antibiotic prophylaxis
LOI: Late-onset infection
MDR: Multidrug resistance
NICU: Neonatal intensive care unit
PAP: Perinatal antibiotic prophylaxis
PFGE: Pulsed-field gel electrophoresis
PROM: Premature rupture of membranes.
